# Halting the Allergic March

**DOI:** 10.1097/WOX.0b013e31816ddbc1

**Published:** 2008-04-15

**Authors:** Hugo P Van Bever, Sudesh T Samuel, Bee Wah Lee

**Affiliations:** 1 Department of Pediatrics, National University Singapore, National University Hospital, 5 Lower Kent Ridge Rd, 119074 Singapore, Singapore

**Keywords:** review, allergy, children, allergic march, probiotics, immunotherapy

## Abstract

The prevalence of childhood allergic diseases, such as allergic asthma, allergic rhinitis, and atopic dermatitis, has increased exponentially. In Singapore, the prevalence of asthma at all ages exceeds 20%, and around 50% of Singaporean children show features of an underlying allergy. The exact environmental causes for the increase of allergic diseases have not yet been identified, but most researchers agree that a decreased bacterial load in young children may be one of the reasons for the increase. However, the causes of allergy are multiple, and the development of an allergic disease is the result of complex interactions between genetic constitution and environmental factors. In this review article, different aspects of allergic sensitization are covered, including prenatal and postnatal sensitization. The phenomenon of the "allergic march" (switching from one clinical expression of allergy to another) and its underlying mechanisms are discussed. The last part of this review article is on prevention and treatment of allergic diseases, including the role of bacterial products (probiotics, prebiotics, and synbiotics) and the role of immunotherapy, including sublingual immunotherapy.

## 

In the past 3 decades, the prevalence of childhood allergic diseases, such as allergic asthma, allergic rhinitis, and atopic dermatitis has increased exponentially. In Singapore, the prevalence of asthma at all ages exceeds 20%, and around 50% of Singaporean children show features of an underlying allergy [1-3]. It is commonly accepted that a child without allergic parents may have up to a 20% risk of developing allergy. If the father (but not the mother) is allergic, the risk is 40%, whereas if the mother (but not he father) is allergic, the risk is 50%. When both parents are known to be allergic, the risk to the child of developing allergy can be as high as 90%. The exact environmental causes for the increase of allergic diseases have not yet been identified, but most researchers agree that a close relationship with a "western lifestyle," one that results in a decreased bacterial load (eg, altered commensal flora) in young children, may be one of the reasons for the increase. This is summarized in the so-called hygiene hypothesis and has been confirmed in studies showing that early contact with bacterial products (eg, from living on a farm, developing infections, attending day care centers) confers a protective effect on the potential development of subsequent allergy [4,5]. In contrast, factors that have been associated with the increased prevalence of allergic diseases include indoor and outdoor pollution, various viral infections (such as infections with respiratory syncytial virus), and an increased use of medication (such as antibiotics and paracetamol) early in life [6,7]. A study from Taiwan has shown that fungi on the walls of the house (adjusted odds ratio [aOR], 2.14; 95% confidence interval, 1.41-3.22) increased the risk of early infantile atopic dermatitis. Fungi might have a role in sensitization, particularly in humid climates where impacts on allergy may be felt at an early infant stage [8].

## Prenatal Sensitization

The cause of allergy is multifactorial, and the development of an allergic disease is the result of complex interactions between genetic constitution and environmental factors. Genetic constitution is important, as it is only in genetically predisposed individuals that the environment is able to trigger symptoms of allergy [9]. In children of parents with asthma, the rate of observed food allergy may be 4 times higher than in the general population [10].

The existence of prenatal sensitization to allergens is still a matter of debate. In some studies, it has been suggested that allergic immune responses start during fetal life, and that the fetus already responds to allergens from week 20 of pregnancy [11]. Fetal exposure to different allergens has been demonstrated from the presence of house-dust mite allergens in amniotic fluid and an active transplacental transport mechanism of different allergens (food allergens and inhalant allergens). The important allergens that have been suggested to cause prenatal sensitization include food allergens (peanuts, eggs, and cow's milk) and inhaled allergens, such as house-dust mites [12,13]. However, from other studies, it seems that cord blood T cells responding to allergens are not memory cells but immature thymic emigrants interacting nonspecifically with allergens, and that these immune reactions are unrelated to subsequent development of allergen-specific T helper cells 2 (Th2) memory or immunoglobulin E (IgE) [14].

Apart from genetic constitution, nutritional, immunologic, and environmental factors acting during pregnancy all play a role in determining whether a child will be born with the propensity to develop allergic sensitization and subsequent allergic disease.

## Postnatal Beginnings

One of the major environmental risk factors that interact with genetic predisposition in the development of allergy is inhaled allergens. However, exposure to certain foreign food proteins, like those found in cow's milk (5%), in early life can also contribute to allergy development in genetically predisposed individuals. In unmodified cow's milk, there are more than 32 types of proteins with potential to trigger food allergy [15]. Similarly, plant proteins, like soy protein, also possess the potential to spark off food allergy. Infants, 15% to 50% of them, who are allergic to cow's milk are also allergic to soya milk [16]. Although any food can cause food allergy, the more potent ones are eggs, milk, peanuts, tree nuts (eg, almond, walnut, cashew, hazelnut, etc), fish, shellfish, and soya bean [17]. Peanut allergy, which affects approximately 0.6% of the general population in the United States, is the most common cause of fatal food-induced anaphylaxis, especially in adolescents with asthma [10]. In Singapore, peanut allergy is less common. The state of the digestive tract and age of the child when first exposed to the offensive food are equally important. Whereas young children and infants may present with eczema, older children can often present with swelling and hives. Sensitivity to most food allergens, such as milk, wheat, and egg, tends to resolve in late childhood, but allergies to peanut, tree nuts, and seafood are likely to be lifelong. Whereas food allergy (egg, cow's milk) is the main type of reaction during the first year of life, allergy to inhaled allergens (house-dust mite, pets) seldom occurs during infancy (although sensitization could already have begun). In young children, eczema and chronic gastroenteritis are usually the first manifestations of allergy, whereas in older subjects, allergy manifests itself more often as a chronic or recurrent respiratory disease (asthma and/or allergic rhinitis).

## The Allergic March

The phenomenon of switching from 1 clinical expression of allergy to another in progressive stages of life is called "the allergic march."[18] Several studies have demonstrated this phenomenon (also known as the atopic march) [19]. In the United Kingdom, Rhodes and colleagues [20] studied 100 infants from atopic families over a 22-year period. The prevalence of atopic eczema reached a peak in 20% of children at 1 year of age and then declined to just below 5% by the end of the study. The prevalence of allergic rhinitis increased from 3% to 15% during the study period. Parent-reported wheezing of the study group increased from 5% during the first year of life to 40% at 22 years of age. Sensitization to allergens as determined by skin-prick tests (SPTs) to 6 common allergens (*Dermatophagoides pteronyssinus*, mixed grasses, dog, cat, egg, and milk) increased to a peak of 36% at 22 years of age. The major risk factor for the development of adult asthma from the study was the early sensitization to either food in the first year of life or aeroallergens in the first 2 years of life. A German study, the large Multicentric Atopy Study, demonstrated the features of the allergic march in 1314 children during a 7-year study period [21]. A high-risk group comprising 38% of the children was identified, where at least 2 family members had atopic diseases or the child had a cord-blood IgE greater than 0.9 kU/L at birth. In this group, 69% of infants who had developed atopic eczema by 3 months of age were sensitized to aeroallergens by the age of 5 years. The rate of aeroallergen sensitization increased to 77% in all high-risk children. At 5 years, 50% of children with early atopic eczema and a positive family history of allergy had developed asthma or rhinitis compared with only 12% of children without eczema or a positive family history of allergy.

In the search for a link between atopic dermatitis and airway allergy, Dohi and colleagues [22] studied 8 patients with bronchial asthma without atopic dermatitis and 8 patients with atopic dermatitis without bronchial asthma for house-dust mite sensitization. The atopic dermatitis patients had a significantly greater concentration of IgE (*P *< 0.01) and antimite IgE antibody (*P *< 0.05) than bronchial asthma patients. Both groups had inhalation challenges to acetylcholine (nonspecific bronchodilator) and to house-dust mites (immunologically specific stimulus). After allergen challenge, all patients showed an immediate asthmatic response, although it was noted that the mite extract concentration required to induce the response was significantly greater (*P *< 0.01) in the atopic dermatitis patients compared with the bronchial asthma patients. A late asthmatic response was also observed in 6 out of 8 bronchial asthma patients compared with none in the atopic dermatitis group, highlighting that a difference in the bronchial reactivity to the allergen between the groups can contribute to a difference in the course of clinical symptoms of asthma. The results suggest that the airways of atopic dermatitis patients are less reactive to a specific mite allergen than bronchial asthma patients despite greater concentrations of antimite IgE antibody.

Further evidence that skin sensitization is linked to airway sensitization was demonstrated when Brinkman and colleagues [23] evaluated the degree of bronchial responsiveness to methacholine in 8 atopic dermatitis patients with allergic asthma and 8 atopic dermatitis patients without allergic asthma followed up with a study of bronchial and cutaneous responses after allergen inhalation challenge. The mean provocative concentration of methacholine causing the same fall in forced expiratory volume in 1 second (FEV_1_) for both groups was significantly higher in the group without allergic asthma. After allergen inhalation challenge, all 8 allergic asthma patients showed early and late asthmatic responses, whereas only 4 patients in the group without allergic asthma showed early asthmatic responses. Although both groups showed cutaneous response after allergen inhalation challenge, the increase in the cutaneous response 24 hours after allergen inhalation challenge was noted to be significantly higher (*P *= 0.016) in the group with allergic asthma. The results show that allergen inhalation can cause a flare-up of skin lesions in atopic dermatitis patients, and this flare-up is likely to be more prominent in those atopic dermatitis patients who already have IgE-mediated allergic inflammation in the lung. It is generally accepted that the increased prevalence of allergic diseases during recent years is caused by a disturbed balance between Th1 and Th2, leading to a greater expression of Th2 features from the secretion of various cytokines that can include interleukin 4 (IL-4), IL-5, IL-6, IL-10, IL-13, and IL-14. These cytokines are able to induce IgE production and activate eosinophils leading to allergic inflammation. The exact reasons for the skewing of the Th1-Th2 balance toward the Th2 profile in allergic individuals are unknown, although decreased postnatal microbial stimulation can result in an increased possibility of ongoing postnatal Th2 reactions.

It is important to address the lead-up to the eventuality of allergic asthma in these children because once asthma sets in, these children (and their parents) are often faced with the significant problems of:

• Trigger avoidance,

• Adherence to anti-inflammatory medication, and

• Stigma in the social context: the challenge for children of not wanting to show that they have asthma. Children may want to hide the fact that they have to take medications and have to avoid trigger factors and become excluded from normal social life.

## Diagnosing Allergy Early

A number of early markers have been identified for atopy, indicating the propensity for the development of the allergic march:

• Elevated cord-blood IgE levels, [24]

• Positive SPTs to egg or house-dust mite in the first year of life, and

• Detection of specific IgE to common foods and inhalant allergens in early infancy.

The use of in vitro panels has proven to be effective in ruling out allergy in children. With sensitivities and negative predictive values exceeding 90%, in vitro panels can provide important information to pediatricians on early diagnosis [25].

The SPT is still considered to be the criterion standard in diagnosing allergy in children of all ages and in adults, but is equally reliable in determining specific IgE in serum. The SPT is prone to human error, and problems with proinflammatory impurities in some SPT allergen preparations can cause potentially false-positive results. Therefore, the accuracy of SPT depends on the skill and experience of the person performing the SPT. Although the sensitivity and specificity of SPT are comparable to those of serum IgE, the SPT offers a number of advantages: faster results (within 30 minutes) and is cheaper than in vitro IgE tests. The SPT can be performed at all ages (even in newborns), is not painful, and is very safe (extremely low risk for any side effect). According to age group, standardized panels of allergens are used based on prevalence of the most common allergens causing allergic disease in Singaporean children. In young children, SPTs are more focused on food allergens (cow's milk, egg), whereas in older children with allergies, inhalant allergens, such as house-dust mites, are used. The SPT can be performed on an outpatient basis. In the future, more sophisticated antibody assay methodologies (such as emerging protein array technology) might become standard of diagnosing allergy.

## Treatment Options

Because allergic diseases are mainly genetically determined, there are no specific cures. However, recent studies on immunotherapy (ie, repeated administrations of well-defined doses of allergen) have shown that this treatment is able to result in sustained desensitization of the patient, even after having stopped the treatment [26]. Immunotherapy, especially the use of sublingual immunotherapy, may be promising for the future, although this type of treatment will only be effective in certain groups of monosensitized patients with rhinitis or asthma and not in all children with allergy (such as children with food allergy or severe eczema) [27].

In recent years, the first studies with anti-IgE (omalizumab) gave promising results, and in 1 study, the combination of anti-IgE and specific immunotherapy was very effective in children with seasonal allergic rhinitis [28]. In most children, however, treatment of allergic diseases mainly constitutes allergen avoidance and symptomatic treatment using corticosteroids, antihistamines, and different antiasthmatic medications.

## Strategies for Prevention

Prevention of allergic diseases and symptoms remains the most attractive approach to date in the battle to halt the allergic march. Some research exists to suggest a benefit of maternal supplementation with omega-3 unsaturated fatty acids (from fish oil) and selenium before delivery [29,30]. Other studies, however, suggest no such effect, and the current overall evidence implies that maternal dietary interventions may not have any preventive effect on the development of allergy. Early prevention often relies on the prevention of sensitization in healthy infants. Such measures include avoidance of early allergen contacts (foods and inhalants) and avoidance of pollution (mainly cigarette smoke). The effect of early allergen avoidance is still a matter of intense debate, and it is still not clear whether primary avoidance of allergens has any beneficial effect or, in contrast, may facilitate allergic sensitization. In a limited number of studies, early avoidance of house-dust mite and pollen has been shown to reduce the occurrence of subsequent allergic diseases. However, in other studies, no effect was observed. In contrast, high concentrations of cat allergen early on seem to induce tolerance [7,31]. As with every infant, breast-feeding is generally considered as first choice for atopic infants, although its potency to inhibit the development of allergy is still unclear. However, most studies agree that, at least, breast-feeding seems to delay or prevent the occurrence of allergy, especially cow's milk allergy [32]. Hypoallergenic formulas have an inhibitory effect on the development of cow's milk allergy. Using an extensively hydrolyzed casein formula has been shown to be of benefit in reducing food allergy and atopic eczema [33].

Infants with atopic dermatitis should be a target group for the prevention of asthma. The late introduction (older than 6 months) of solid foods also seems to be advisable in atopic infants, whereas administration of eggs should be avoided in infants with moderate to severe atopic dermatitis [34].

At this time, a limited amount of data is available concerning the preventive effect of medication on the development of allergic diseases. In a limited number of studies, the early administration of ketotifen in infants with eczema was able to prevent the occurrence of asthma. In a separate study, the same drug inhibited the occurrence of asthma in high-risk symptom-free infants. Iikura and colleagues [35] treated 121 children with atopic eczema (aged 1-36 months) with either ketotifen or placebo before the onset of asthma. After 1 year of study, significantly fewer patients in the ketotifen group had developed asthma compared with the placebo group.

More recently, the Early Treatment of the Atopic Child Study involving 795 European children found that cetirizine, administered for 18 months, was able to delay or prevent the development of asthma in young children with atopic eczema and who were allergic to house-dust mite (51% placebo vs 28.6% cetirizine developed asthma) and/or grass pollen (58% placebo vs 27.8% cetirizine developed asthma) [36,37]. However, in a second large study on levocetirizine known as the Early Prevention of Asthma in Atopic Children Study, such results were not confirmable. The Early Prevention of Asthma in Atopic Children Study and follow-up examined the role of levocetirizine in delaying the allergic march. This was a prospective, randomized, double-blind, parallel group, and placebo-controlled study that enrolled 2500 children aged 1 to 2 years in Europe, South Africa, and Australia. The children had atopic eczema and a family history of atopy. The treatment duration was prolonged to 3 years in total, and specific IgE levels were measured at baseline and at regular intervals throughout the study period. By the end, 40% of the infants had developed asthma and results showed that children with early sensitization to egg, milk, cat, grass, or house-dust mite had an increased risk of developing asthma. The study showed no significant difference between the levocetirizine and placebo-treated groups in the development of asthma. The role of prebiotics, synbiotics, and probiotics remains unclear, and more studies are needed before general recommendations can be given. Prebiotics are nondigestible fermentable oligosaccharides that stimulate the growth of *Bifidobacterium *and *Lactobacillus *species [38]. Altering the intake of foods containing these products can directly influence the composition and activity of intestinal microbiota, and this could explain some of the protective effects of grains and cereals that have been observed in epidemiological studies [39]. There remains, however, very little evidence of efficacy in the use of such therapy. Probiotics are live microbial food supplements that are believed to beneficially and safely alter the intestinal microbial balance [38]. Synbiotics are a combination of prebiotics and probiotics. The gastrointestinal tract functions as a barrier against antigens from microorganisms and food, and specific strains of healthy gut microbiota may aid in regulating the secretion of inflammatory mediators and in directing immune system development during the period of life when the risk of allergic disease is very high. From studying mice, at least some of the anti-inflammatory effects seem to be mediated through toll-like receptor 9 (TLR9) and possibly TLR2 and TLR4 expressed on enterocytes [40]. Intestinal microbiota also promotes enterocyte production of tumor necrosis factor-β and prostaglandin E2, which promote the development of tolerogenic dendritic cells [41].

The basis for using probiotics in allergic disease stems from several observations [38]:

• Epidemiological links between less exposure to microbes and rising incidence of allergy

• Intestinal microbiota--largest source of microbial exposure in early immune development

• Intestinal microbiota seems essential for development of oral tolerance

• Changing patterns of microbiota with progressive westernization

• Differences in perinatal colonization in children who go on to develop allergic disease

• Perinatal probiotic use associated with anti-inflammatory immunomodulatory effects

• Presymptomatic immune dysregulation in infants and newborns predisposed to allergy

• Initial studies with probiotics suggested promise in prevention and early treatment

A study that looked at allergy development and intestinal flora during the first year of life found that infants who developed allergies often did not have significant levels of enterococci colonization during the first month after birth (72% vs 96% in healthy infants). In addition, infants with allergies were also less apt to be colonized with bifidobacteria during their first year (17%-39% vs 42%-69% in healthy infants). It seems that early administration of probiotics (starting during pregnancy), in combination with breast-feeding, is able to reduce the occurrence of eczema [42]. *Lactobacillus*, given during pregnancy and to infants for 6 months may offer protective benefits against atopic dermatitis for the first 2 years of life [28,29]. The beneficial effects of probiotics may, however, be limited by species. In 1 study, *Lactobacillus acidophilus *failed to show any reduction in allergic disease, despite changes in colonization [43]. A recent study showed that *Lactobacillus reuteri *and *Lactobacillus casei*, but not *Lactobacillus plantarum*, primed monocyte-derived dendritic cells to drive the development of regulatory T cells (more Th1) [44]. Further studies have pointed to beneficial effects with species that include *Lactobacillus GorbachGoldin, Lactobacillus rhamnosus*, and *Lactobacillus fermentum*, along with *Bifidobacterium lactis*. One study showed that a combination of strains and prebiotic galactooligossaccharides led to a reduction in atopic eczema, but had no effect on sensitization or other allergic disease [39]. Of note, however, is that most of these studies either had small numbers and/or infants and children with mild disease less likely to embark on the allergic march [38]. In 1 study on *L. Gorbach Goldin*, the beneficial effects were only observed in children with evidence of allergic sensitization and not in children with atopic dermatitis but no sensitization in those who received the same probiotics. This finding highlights the heterogeneity of atopic dermatitis and the fact that the pattern of the disease can significantly affect the effect of supposedly beneficial probiotics. Also of note is the lack of effect of such probiotics in older individuals with asthma and allergic rhinitis, suggesting that any beneficial effects may be confined to early life before allergic disease becomes established. Despite a sound theoretical basis for anticipating benefits, there are insufficient data to recommend probiotics as a part of standard therapy in any allergic condition [38]. Furthermore, despite several studies showing benefit in prevention of atopic eczema, other studies fail to support this. Strains used are also important as the term *probiotic *is also often used loosely to include bacterial strains with little documented immunomodulatory capacity or controlled studies to support claims. It is unlikely that supplementation with a single probiotic strain is sufficient to have a major influence on the diversity of intestinal microbiota. The complex interactions between gut bacteria and host encourage an orientation toward altering dietary substrates as with prebiotics in the hope that this will have a more global effect on gut microbiota.

## Conclusions

Allergic diseases have now become the most common group of diseases among children, affecting up to 50% of children in the Singaporean population. Treatment is mainly based on preventing and/or controlling symptoms. New promising treatments have become available, such as the administration of sublingual immunotherapy. In the future, it is anticipated that therapies that modify the severity of atopic eczema in infants and young children will decrease the risk for the eventual development of asthma and thus prevent the consequences of the allergic march. Intensive research is still required before a potential cure for all allergic children may eventually be developed.
